# Efficacy and Underlying Mechanism of Acupuncture in the Treatment of Posttraumatic Stress Disorder: A Systematic Review of Animal Studies

**DOI:** 10.3390/jcm10081575

**Published:** 2021-04-08

**Authors:** Chan-Young Kwon, Boram Lee, Sang-Ho Kim

**Affiliations:** 1 Department of Oriental Neuropsychiatry, Dong-Eui University College of Korean Medicine, Busan 47227, Korea; beanalogue@deu.ac.kr; 2 Clinical Medicine Division, Korea Institute of Oriental Medicine, 1672 Yuseongdae-ro, Yuseong-gu, Daejeon 34054, Korea; qhfka9357@kiom.re.kr; 3 Department of Neuropsychiatry of Korean Medicine, Pohang Korean Medicine Hospital, Daegu Haany University, 411 Saecheonnyeon-daero, Nam-gu, Pohang-si 790-826, Korea

**Keywords:** post-traumatic stress disorder, acupuncture, East Asian traditional medicine, animal studies, mechanisms

## Abstract

Acupuncture is a nonpharmacological intervention that can be useful in the clinical management of posttraumatic stress disorder (PTSD), especially in situations with a lack of medical resources, including large-scale PTSD events such as disasters. Some clinical studies have reported the clinical effect of acupuncture in improving PTSD symptoms, but the underlying therapeutic mechanism has yet to be explored. Therefore, this review summarized the underlying therapeutic mechanisms of acupuncture in animal PTSD models. A comprehensive search was conducted in 14 electronic databases, and two independent researchers performed study selection, data extraction, and the methodological quality assessment. Twenty-four relevant studies were included in this review and summarized according to the proposed main mechanisms. In behavioral evaluation, acupuncture, including manual acupuncture and electro-acupuncture, reduced anxiety and fear responses and weakened fear conditioning, improved sleep architecture, reduced depressive symptoms, and alleviated disturbance of spatial learning and memory of PTSD animal models. The therapeutic mechanisms of acupuncture proposed in the included studies could be classified into two categories: (1) regulation of stress responses in the neuroendocrine system and (2) promotion of neuroprotection, neurogenesis, and synaptic plasticity in several brain areas. However, the methodological quality of the included animal studies was not high enough to produce robust evidence. In addition, mechanistic studies on specific aspects of acupuncture that may affect PTSD, including expectancy effects, in human PTSD subjects are also needed.

## 1. Introduction

Direct or indirect exposure to exceptionally threatening or terrifying events can lead to a debilitating mental disorder, i.e., posttraumatic stress disorder (PTSD). Characteristic signs and symptoms of PTSD include intrusion symptoms, avoidance, negative alterations in cognitions and mood, and alterations in arousal and reactivity [1]. In primary care, the prevalence of this disorder is common, and a systematic review in 2017 found that the lifetime prevalence of PTSD confirmed by diagnostic interviews ranged from 2% to 39.1%, with a median point prevalence of 12.5%, similar to that of depression [2]. Studies have shown that risk factors for PTSD include witnessing someone being killed, having no regular income after an earthquake, lower social support, drug and/or alcohol abuse, current smoking, history of chest pain, being female, low educational or socioeconomic status, and prior psychological trauma [3,4,5].

For the treatment of PTSD, some pharmacotherapies, such as fluoxetine, paroxetine, sertraline, and venlafaxine, may be effective, while current clinical practice guidelines strongly recommend psychological therapies, such as cognitive-behavioral therapy, cognitive processing therapy, cognitive therapy, prolonged exposure therapy, brief eclectic psychotherapy, eye movement desensitization and reprocessing, and narrative exposure therapy [6]. However, the high human resource dependence of psychotherapies and the limited efficacy of pharmacotherapies have become challenges in large-scale PTSD events such as disasters.

Acupuncture is a nonpharmacological therapy originating in East Asia but is now being used worldwide. The use of acupuncture has been investigated not only in various pain conditions [7] but also in psychiatric problems such as depression [8], insomnia [9], and anxiety disorders [10]. More robust evidence is needed to be recognized as an evidence-based treatment for PTSD [11], but acupuncture is a promising complementary and alternative medicine approach for PTSD symptoms [12,13]. Accordingly, the authors reviewed the possibility that ear acupuncture could be used for the treatment and management of PTSD in disaster sites as an effective alternative approach [14]. However, the clinical evidence supporting the use of acupuncture in PTSD still needs to be strengthened, and the therapeutic mechanism of acupuncture needs to be further elucidated. Although a review in 2011 by Hollifield M [13] indicated that biological mechanisms such as improvements in alterations in central nervous system improvement, hypothalamic-pituitary-adrenal (HPA) dysfunction, and autonomic nervous system dysfunction may be related to the effects of acupuncture on PTSD, the preclinical evidence involved has not been comprehensively synthesized.

A systematic review of preclinical animal studies can improve translational medicine and potentially lead to more accurate health care decisions [15]. In addition, understanding the therapeutic mechanisms of acupuncture as well as related outcomes derived from preclinical animal studies can help establish relevant biomarkers for acupuncture in human subjects with PTSD. In PTSD, some models, such as single-prolonged stress (SPS), restraint stress, foot shock, stress-enhanced fear learning, underwater trauma, predator-based psychosocial stress/predator scent stress, housing instability, social instability, early life stress, and social defeat, have been used to mimic human PTSD [16]. Moreover, when the results of these animal experiments can be translated into human subjects, it could potentially improve the clinical aspects of PTSD [16]. Therefore, in this systematic review, the authors attempted to uncover the underlying mechanisms of acupuncture, including manual acupuncture (MA) and electro-acupuncture (EA), in PTSD animal models. Furthermore, the authors attempted to discuss the implications for clinical research of acupuncture on PTSD in the future based on this finding.

## 2. Materials and Methods

This systematic review followed the Preferred Reporting Items for Systematic Reviews and Meta-Analyses (PRISMA) statement [17] and the methodology of the Cochrane Handbook for Systematic Reviews of Interventions version 5.1.0 [18] (Appendix A).

### 2.1. Search Strategy

The following English, Korean, Chinese, and Japanese electronic databases were searched from inception to 18 March 2020 without restrictions regarding publication status and language by one researcher (BL): Medline, EMBASE, the Allied and Complementary Medicine Database (AMED), Cumulative Index to Nursing and Allied Health Literature (CINAHL), PsycARTICLES, Oriental Medicine Advanced Searching Integrated System (OASIS), Koreanstudies Information Service System (KISS), Research Information Sharing Service (RISS), Korean Medical Database (KMbase), Korea Citation Index (KCI), China National Knowledge Infrastructure (CNKI), Wanfang data, VIP, and CiNii. The search keywords used were “posttraumatic stress disorder”, “acupuncture”, and “animal”. We also searched the reference lists of the included studies to identify additional eligible studies. The following keywords in the title and abstract were used in the search strategy: posttraumatic stress disorder, PTSD, acupuncture, and electroacupuncture. The detailed search strategies used in all databases are presented in Appendix B.

### 2.2. Inclusion Criteria

Randomized controlled experimental studies investigating the effect of acupuncture in animal models of PTSD were included. We included all studies using MA and EA as experimental interventions regardless of the acupuncture stimulation method, and there were no restrictions regarding the duration and number of acupuncture treatments or the type of control interventions. Moreover, there was no language restriction. However, as the authors are fluent only in the Korean, English, Japanese, and Chinese languages, if there were papers published in other languages, the authors planned to receive professional translation services from an academic service company.

### 2.3. Study Selection and Data Extraction

We used EndNote X8 (Clarivate Analytics, Philadelphia, PA, USA) to import the retrieved articles from the abovementioned databases and other sources. Then, we deleted duplicate articles and screened the titles and abstracts. Eligibility was confirmed by reviewing the full text of suitable studies. The following data were extracted from the included studies: (1) basic information such as the name of the first author, publication year, and country, (2) characteristics of animal models including species, sex, age, weight, and the method of establishing the PTSD model, (3) details of the acupuncture method, outcome, and results, and (4) the proposed mechanism in the included studies, and (5) funding sources of the included studies. Two researchers (CYK and BL) independently performed the study selection and data extraction process, and any disagreements were resolved consulting the third researcher (SHK).

### 2.4. Quality Assessment

The risk of bias of the included studies was assessed using Systematic Review Centre for Laboratory Animal Experimentation (SYRCLE)’s risk of bias tool [19]. This tool contains 10 items related to selection bias (sequence generation, baseline characteristics, and allocation concealment), performance bias (random housing and blinding), detection bias (random outcome assessment and blinding), attrition bias (incomplete outcome data), reporting bias (selective outcome reporting), and other biases (any important concerns about bias not covered by other domains). Each item was evaluated as three categories: ‘Yes’, ‘No’, and ‘Unclear’. Two researchers (CYK and BL) independently evaluated the risk of bias for the included studies, and any disagreements were resolved through discussion with a third researcher (SHK). Review Manager version 5.3 software (Cochrane, London, UK) was used to generate the risk of bias figure.

### 2.5. Data Analysis

The descriptive analysis of basic information, characteristics of animal models, acupuncture method, outcome, results, and the proposed mechanism of acupuncture for each included study was conducted. Due to the significant clinical heterogeneity between the studies included, quantitative synthesis was not performed.

## 3. Results

### 3.1. Search Results

**Figure 1:**
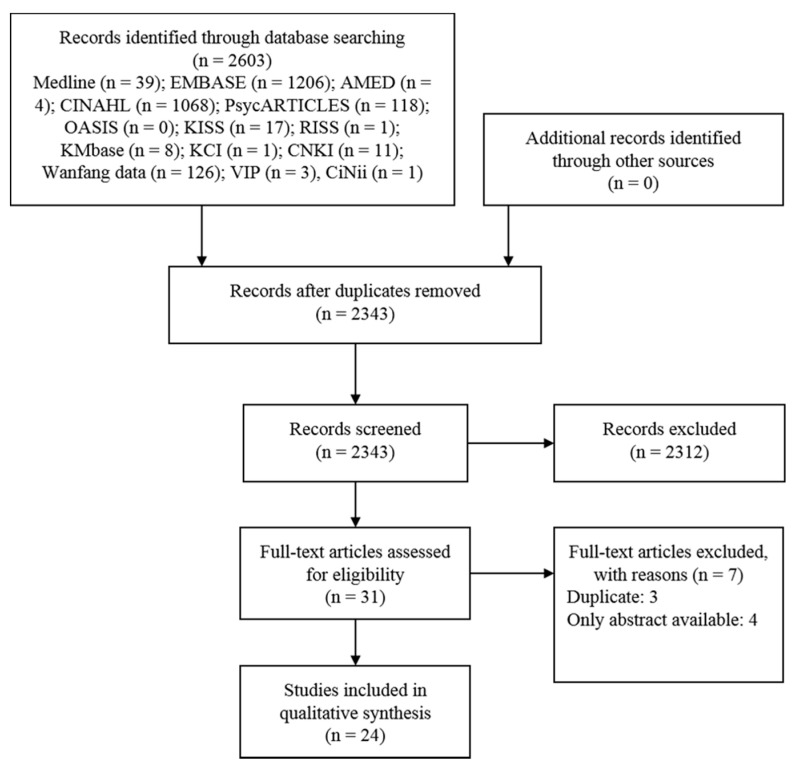
A PRISMA flow diagram of the literature screening and selection processes. AMED, Allied and Complementary Medicine Database; CINAHL, Cumulative Index to Nursing and Allied Health Literature; CNKI, China National Knowledge Infrastructure; KCI, Korea Citation Index; KISS, Korean Studies Information Service System; KMbase, Korean Medical Database; OASIS, Oriental Medicine Advanced Searching Integrated System; RISS, Research Information Service System.

A total of 2603 studies were identified from the databases, and there were no studies from other sources. Duplicate studies were removed, and then, another 2312 articles were excluded through title and abstract screening. During the process of reviewing the full texts of the remaining 31 articles, three duplicate articles [20,21,22] and four studies with abstracts but no full texts [23,24,25,26] were excluded. Therefore, a total of 24 studies [27,28,29,30,31,32,33,34,35,36,37,38,39,40,41,42,43,44,45,46,47,48,49,50] were included in this review (Figure 1).

### 3.2. Study Description

Twenty-three studies were conducted in China, and the remaining study was performed in the Republic of Korea [44]. All studies were published in Chinese or English. With the exception of two studies [30,32] without mention of funding sources, all remaining 22 studies were funded by national, provincial, or postdoctoral research funds (Appendix C). Regarding the species of rats, 23 studies [27,28,29,30,31,32,33,34,35,36,37,38,39,40,41,42,43,44,45,46,47,48,50] used SD rats, and one [49] used Wistar rats. Regarding sex, 23 studies [27,28,29,30,31,32,33,34,35,36,37,38,39,40,42,43,44,45,46,47,48,49,50] used male rats, and the remaining study [41] did not provide sex-related data. Of the 12 studies [33,35,36,42,43,44,45,46,47,48,49,50] that presented the age of rats, 8 studies [33,35,36,42,44,48,49,50] used rats that were 8 weeks or 2 months old, and 4 studies [43,45,46,47] used rats that were 6 weeks old. The weight of the rats varied from 180 to 320 g. A total of 3 kinds of PTSD models were used: SPS model was used in 12 studies [27,28,29,31,32,35,37,38,39,40,41,44], a single-prolonged stress accompanied shock (SPS&S) model was used in 10 studies [33,34,42,43,45,46,47,48,49,50], both SPS and SPS&S models were used in 1 study [36], and a compound stress model of restraint, electric shock, and exhaustive swimming was used in 1 study [30] (Table 1).

A total of 26 experiments were conducted across 24 studies because 2 studies [42,44] using MA conducted two experiments. For the acupuncture stimulation method, 15 articles [27,28,29,30,31,32,33,34,35,36,37,38,39,40,41] used EA, and the remaining 9 studies with 11 experiments [42,43,44,45,46,47,48,49,50] used MA. A total of 10 acupoints were used in all included studies. Among them, Baihui (GV 20) was the most frequently used acupoint in 23 experiments [27,28,29,30,31,32,33,34,35,36,38,39,40,41,42,43,45,46,47,48,49,50], followed by Neiguan (PC 6), Shenmen (HT 7), and Taichong (LR 3) in 9 experiments [42,43,45,46,47,48,49,50]. For needle retention time in experiments using EA, 20 min [30,31,34,36,37,38,41] and 30 min [28,29,32,33,35,39,40] were the most common durations (7 experiments), followed by 15 min (1 experiment) [27]. In experiments using MA, 9 experiments across 8 studies [42,43,45,46,47,48,49,50] retained the needle for 4 min, and 2 experiments in 1 study [44] did not retain it. Acupuncture was performed once a day in all studies, and the total number of acupuncture treatments varied from 3 to 21 sessions, of which 7 sessions was the most common (10 experiments) [28,29,31,33,35,37,38,39,40,49]. The treatment period also varied from 3 to 21 days, of which one week was the most common in 11 experiments [28,29,31,32,33,35,37,38,39,40,49] (Appendix D).

### 3.3. Methodological Qualities of Included Studies

**Figure 2:**
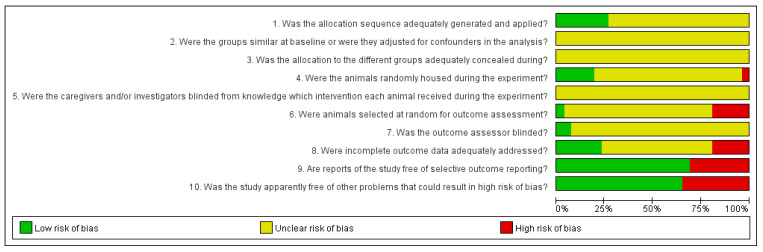
SYRCLE results of included animal studies.

For the sequence generation domain, 7 experiments in 6 studies [42,43,46,48,49,50] were rated as having a low risk of bias (“Yes”) because they used appropriate random sequence generation methods, such as computer-generated random numbers. The remaining 19 experiments [27,28,29,30,31,32,33,34,35,36,37,38,39,40,41,44,45,47,48,50] were rated as having an unclear risk of bias because they did not describe the method. For baseline characteristics, allocation concealment, and blinding domains, all studies rated them as having an unclear risk of bias because they had no information regarding them. For the random housing domain, 5 experiments in 4 studies [37,44,48,50] randomly housing animals during the experiment were evaluated as having a low risk of bias, and 1 experiment [46] assigning cages according to groups was assessed as having a high risk of bias (“No”). One experiment [46] selecting animals at random for outcome assessment and 2 experiments [34,37] conducting blinding of outcome assessor were assessed as low risk of bias in random outcome assessment and outcome assessor blinding domains, respectively. Five experiments [42,43,44,45] that did not include all of the animals tested in the analysis were evaluated as having a high risk of bias in the incomplete outcome data domain. Eight experiments [28,30,41,44,46,47,48,50] that did not mention any experimental results or relevant mechanisms were evaluated as having a high risk of bias in the selective outcome reporting domain. Nine experiments [27,28,30,31,32,33,35,39,40] were evaluated as having a high risk of bias in other sources of bias domain because acupuncture treatment methods were not described in sufficient detail (Figure 2 and Appendix E).

### 3.4. Main Results for Each Symptom of PTSD

#### 3.4.1. Anxiety & Fear

A total of nine studies [27,32,33,34,35,36,37,39,40] targeted anxiety or fear symptoms of PTSD by using EA. Among them, 8 studies [27,32,33,35,36,37,39,40] conducted an elevated plus-maze (EPM) test, and all studies showed that the EA group spent significantly more time in the open arms and more entries in the open arms than the model group, meaning reduced anxiety levels. Five studies [33,35,37,39,40] conducted open field tests (OFTs), and all studies reported that the EA group had significantly more entries in the center and more distance traveled in the center than the model group, although there was no difference between the two groups in total distance. In the case of time spent in the center, there was inconsistent results between studies, showing results in favor of the EA group in 4 studies [33,37,39,40] and no difference between the two groups in 1 study [35]. Two studies [39,40] conducted fear conditioning tests, and the contextual freezing time and cued freezing time were significantly shorter in the EA group than in the model group. One study [34] assessed scene- and cue-conditioned fear response detection, and the results showed that memory extinction and reconstruction of each had significantly lower values in the EA group, although there was no significant difference between groups in memory acquisition. One study using MA [44] conducted the EPM test and showed that entries and time spent in open arms and the anxiety index were significantly higher in the MA group than in the model group, although there was an inconsistent result in entries in closed arms. Three studies [43,44,47] performed OFT and showed that there were favorable results to the MA group in entries in the center, horizontal crossing grid number, vertical frequency, and the number of fecal particles. However, there were inconsistent results in travel distance between the two groups.

#### 3.4.2. Sleep Disturbance

One study [38] assessed sleep disturbance in PTSD using EA. There were significantly higher values of rapid eye movement sleep (REMS) and slow wave sleep stage 2 in the EA group than in the model group, although there was no difference in slow wave sleep stage 1 between the two groups. Two studies [42,49] assessed sleep disturbance in a PTSD model using MA. They showed that non-REMS and REMS latency and awakening period were significantly shorter in the MA group than in the model group. In addition, the total sleep time was significantly longer in the MA group.

#### 3.4.3. Cognitive Symptom

Two [29,30] targeted spatial learning and memory symptoms of PTSD using EA. They [29,30] performed the Morris water maze (MWM) test, and the escape latency was significantly shorter in the EA group than in the model group. Three studies [43,45,47] targeted cognitive symptoms by conducting the MWM test using MA. In the MA group, the daily latency during the positioning navigation experiment was significantly shorter, and the space exploration experiment times crossing the platform or effective areas were significantly longer than those in the model group.

#### 3.4.4. Depression

One study using MA [44] included a forced swim test (FST) to test the depression level, and the results showed that immobility time was significantly shorter and climbing time was significantly longer in the MA group than in the model group, although there was no difference between the two groups in swimming time.

### 3.5. Proposed Mechanisms

#### 3.5.1. Regulation of Stress Responses in the Neuroendocrine System

The altered function of the HPA axis has a significant impact on the pathology of PTSD and can all be related to stress reactivity, fear learning and extinction, and inflammation [51]. A study using EA suggested the mechanism of acupuncture against PTSD as downregulation of HPA axis activity [27]. Specifically a study found that EA regulated the levels of tumor necrosis factor (TNF)-α and interleukin (IL)-4 in the hypothalamus of the PTSD animal model [38]. Moreover, for the locus coeruleus, which plays an important role in stress responsiveness as a nucleus in the brainstem [52], one study reported that EA can reduce excessive stress responses of PTSD model by downregulating nitric oxide synthase (nNOS) expression in this region [31]. Finally, one study [29] reported upregulation of hippocampal mineralocorticoid receptor expression and downregulation of hippocampal glucocorticoid receptor expression after EA. Both are adrenal steroid receptors that affect the HPA axis through negative glucocorticoid feedback [53]. Importantly, the activation of these receptors is related to memory, behavioral responsibility, anxiety, and fear [54]. Moreover, another study [28] reported downregulation of hippocampal neuronal nNOS expression, which regulate the behavioral effects of glucocorticoids [55], after EA. These results suggest the role of acupuncture in neuroendocrine regulation.

#### 3.5.2. Promotion of Neuroprotection, Neurogenesis, and Synaptic Plasticity in Brain

(1) Hippocampus: In PSTD, the hippocampus forms a link between situational stimuli and aversive events and plays an important role in memory recall [56]. Thus, hippocampal dysfunction is considered an important factor that causes PTSD or maintains it by interacting with traumatic experiences [56]. Furthermore, since chronic stress is known to cause atrophy of the hippocampus, interventions targeting adult neurogenesis and synaptic plasticity improvement in the hippocampus may be useful for PTSD and other mental disorders [57]. In this respect, several studies included in this review have reported the potential impact of acupuncture on the hippocampus: upregulation of thioredoxin (Trx) expression in the hippocampus [32], restoration of messenger ribonucleic acid (mRNA) levels of sirtuin 1 and monoamine oxidase A in the hippocampus [33], improvement and repair of synaptic plasticity in hippocampus [36], improvement of hippocampal neurogenesis [40], upregulation of the protein synthesis required for synaptic plasticity via the mammalian target of rapamycin pathway in the hippocampus [44], restoration of the structure of hippocampal neuronal cells [42,49], reverse of the discharge activity of neurons in the hippocampus [49], and regulation of abnormal neuronal cluster electrical activity in the hippocampal CA1 and CA3 regions [48,50].
(2) Amygdala: In addition to the hippocampus, the amygdala is the most clearly altered limbic region in PTSD, which can lead to an overactive reaction to fear stimuli [58]. In the fear circuit, the hippocampus is thought to play an important role in the explicit memory of traumatic events and mediate learned responses to contextual cues. The PFC reactivates past emotional associations, and the amygdala is activated in PTSD subjects and associated with oversensitivity to stress, generalized fear responses, and impaired extinction [58]. Thus, interventions that promote synaptic plasticity in the amygdala could potentially contribute to fear control and extinction [58]. In this respect, some studies included in this review have reported on the potential impact of acupuncture on the amygdala: upregulation of brain-derived neurotrophic factor (BDNF), tropomyosin-related kinase B (TrkB), phosphorylated mitogen-activated protein/extracellular signal-regulated kinase (p-MEK), phosphorylated extracellular signal-regulated kinase (p-ERK) expression in the amygdala [34], prevention of increase in tyrosine hydroxylase (TH) level and decrease in BDNF level in the amygdala [41], and improvement and repair of synaptic plasticity in the amygdala [36].
(3) Anterior cingulate cortex: In the anterior cingulate cortex (ACC), c-Fos expression is related to emotional regulation and attention and cognitive control [59,60], which are all related to major pathologies of PTSD. c-Fos is a gene involved in cell proliferation and differentiation after extracellular stimuli [61]. In ACC, which plays a role in regulating conditioned fear responses [62], c-FOS expression was significantly increased after EA [37].
(4) Prefrontal cortex: Endocannabinoids regulate various forms of synaptic plasticity in the adult brain, and enhancement of cannabinoid type 1 receptor (CB1R) is highly expressed in the forebrain [63]. A study found that EA could upregulate expressions of CB1R and diacylglycerol lipase α (DAGLα), which generate 2-arachidonoylglycerol (the most abundant ligand for the CB1R), in the PFC [35].
(5) Others: One study [46] indicated the regulatory effect of MA on the blood oxygen level in the cerebral cortex.

## 4. Discussion

**Figure 3:**
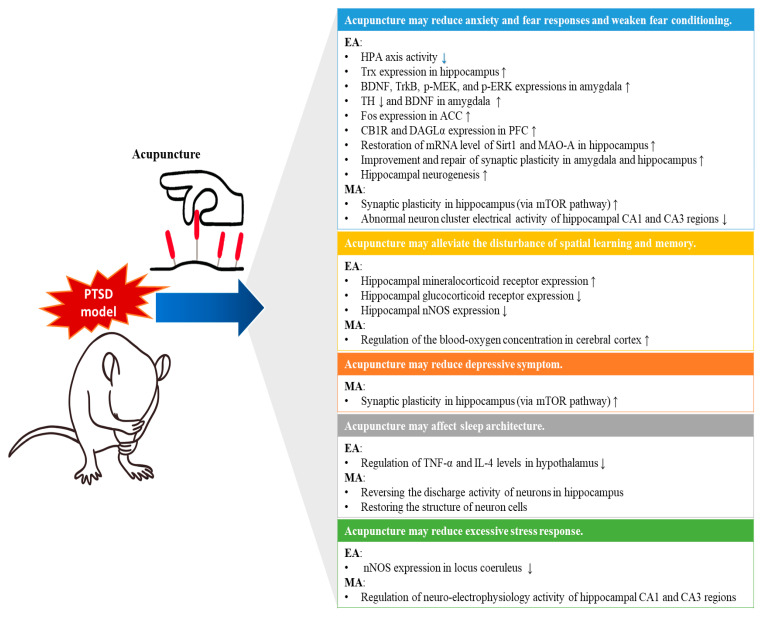
Possible underlying mechanisms of acupuncture for PTSD. ACC, anterior cingulate cortex; BDNF, brain-derived neurotrophic factor; CB1R, cannabinoid type 1 receptor; DAGLα, diacylglycerol lipase α; EEG, electroencephalogram; HPA axis, hypothalamic-pituitary-adrenal axis; IL, interleukin; MAO-A, monoamine oxidase A; mRNA, messenger ribonucleic acid; mTOR, mammalian target of rapamycin; nNOS, neuronal nitric oxide synthase; p-ERK, phosphorylated extracellular signal-regulated kinase; p-MEK, phosphorylated mitogen-activated protein/extracellular signal-regulated kinase kinase; PFC, prefrontal cortex; Sirt1, Sirtuin 1; TH, tyrosine hydroxylase; TNF, tumor necrosis factor; TrkB, tropomyosin-related kinase B; Trx, thioredoxin.

In this review, previously investigated therapeutic mechanisms of acupuncture for various symptoms of PTSD were collected and analyzed from preclinical studies. Through comprehensive literature searches, 24 relevant animal studies [27,28,29,30,31,32,33,34,35,36,37,38,39,40,41,42,43,44,45,46,47,48,49,50] were included in the analysis. All but one study used the SPS and/or SPS & S model to establish the PTSD model, which is a representative model that mimics the pathology of human PTSD [64], and it is considered to faithfully reflect the PTSD criteria presented in the Diagnostic and Statistical Manual of Mental Disorders (DSM)-5 [16]. One study [30] that used the compound stress model of restraint, electric shock, and exhaustive swimming is also considered to reflect the DSM-5 criteria [16]. Therefore, PTSD models of all included animal studies can be considered reliable and potentially relevant to human outcomes. In these studies, the effects and/or underlying mechanisms of acupuncture on anxiety and fear behaviors, depression behavior, sleep disturbance, cognitive symptoms (especially spatial learning and memory), and stress responses in PTSD animal models have been reported (Figure 3 and Table 2).

In behavioral evaluation, acupuncture, including MA and EA, reduced anxiety and fear responses and weakened fear conditioning, improved sleep architecture, reduced depressive symptoms, and alleviated the disturbance of spatial learning and memory in PTSD animal models. The therapeutic mechanisms of acupuncture proposed in the included studies could be classified into two categories: (1) regulation of stress responses in the neuroendocrine system and (2) promotion of neuroprotection, neurogenesis, and synaptic plasticity in several brain areas.

### 4.1. Regulation of Stress Responses in the Neuroendocrine System

In PTSD animal models, acupuncture modulated the reactivity of the HPA axis, including reduction of inflammation in the hypothalamus. The axis plays an extremely important role in stress management, and chronic stress or psychological trauma can lead to pathological consequences of this axis. Although the relevance of dysregulated HPA axis functionality in patients with PTSD has not yet been firmly proven [65], the HPA axis-modulating effect of acupuncture treatment may also gain more attention given that the HPA axis cannot be overlooked in the pathology of PTSD. Moreover, anti-inflammatory effects in the hypothalamus may be related to the observed improvement of sleep architecture and even memory consolidation. Disruption of sleep architecture is a common clinical manifestation of PTSD, and some sleep stages, especially non-REM slow wave sleep, play an important role in memory consolidation and may affect other symptoms of PTSD [66]. In addition, disturbed REMS or non-REMS may contribute to maladaptive stress and trauma responses [67]. Although there is limited certainty, there is some evidence that acupuncture can improve sleep quality in patients with insomnia [68]. In a recent randomized controlled trial [69], acupuncture significantly decreased non-REMS stages 1 and 2, increased non-REMS stage 3, and improved episodic memory in patients with chronic insomnia disorder. Given these results, acupuncture may improve sleep quality for PTSD and indirectly affect other symptoms of PTSD, such as memory consolidation. Neuroinflammation is a promising research field in PTSD [70], and some studies have reported that acupuncture reduces neuroinflammation in areas of the brain that play a key role in sleep regulation, such as the hypothalamus [42,49], but further studies are needed. As other aspects of the stress response, acupuncture downregulated nNOS expression in the locus coeruleus and hippocampus, which are associated with stress responses, upregulation of hippocampal mineralocorticoid receptor expression, and downregulation of hippocampal glucocorticoid receptor expression. These results suggest the role of acupuncture in the regulation of stress responses by neuroendocrine regulation.

### 4.2. Promotion of Neuroprotection, Neurogenesis, and Synaptic Plasticity in Several Brain Areas

Acupuncture has been reported to promote neuroprotection, neurogenesis, and synaptic plasticity in several areas of the brain in the PTSD model. In the pathogenesis of PTSD, BDNF has been suggested to be responsible for fear learning by controlling fear circuit plasticity [71]. The main areas associated with anxiety and fear responses in this hypothesis include the hippocampus, PFC, and amygdala [71]. In the fear circuit, the hippocampus is thought to play an important role in the explicit memory of traumatic events and mediate learned responses to contextual cues; the PFC reactivates past emotional associations; and the amygdala is activated in subjects with PTSD and associated with oversensitivity to stress, generalized fear responses, and impaired extinction [58]. Among these, the hippocampus and amygdala play a key role in conditioned fear and associative learning [58]. According to our findings, acupuncture is thought to modulate neuroplasticity in several brain regions, including hippocampus, amygdala, ACC, and PFC. Particularly, effects on the hippocampus have been reported most often, suggesting that acupuncture may have the potential to reduce anxiety and fear symptoms by controlling the fear circuit plasticity of PTSD, which is related to conditioned fear and associative learning clinically.

### 4.3. Comparison with Previous Clinical Studies

The findings of this review suggest promising potentials of acupuncture for treatment of PTSD at the preclinical level. However, reproducibility in clinical studies involving human PTSD subjects is considered highly insufficient. For example, a systematic review that evaluated the effectiveness of acupuncture on PTSD, including seven randomized controlled trials of 3–12 weeks, not only pointed out extremely low quality of the body of evidence (QoE) but also no significant difference on depressive symptoms, anxiety symptoms, and sleep quality between the acupuncture and control groups, at post-treatment [11]. However, although the QoE was low, significant improvement in PTSD and depressive symptoms was observed in longer follow-up (1 to 6 months post-intervention) [11]. There are also differences arising from the limitations of animal studies. For example, a systematic review was published in 2017 reporting the effectiveness of emotional freedom techniques (EFT) using cognitive and exposure therapies simultaneously with manual stimulation of acupoints for PTSD [72]. At the same time, a study published in 2018 concluded that manual stimulation of acupoints during EFT could not be explained by just placebo or nonspecific effects [73]. Thus, EFT includes aspects of cognitive therapy that are difficult to experiment in animal experiments, and stimulation of acupoints is thought to have a unique therapeutic effect. Moreover, although the level of evidence is very insufficient [74], factors such as patient-practitioner interactions are likely to influence the clinical outcome of acupuncture [75]. Therefore, the findings of this review may not fully support several acupoint-based interventions for human patients with PTSD.

### 4.4. Strengths and Limitations of this Review

Acupuncture is a nonpharmacological intervention that can be useful in the clinical management of PTSD, especially in situations where medical resources are prone to lack, including large-scale PTSD events such as disasters. However, since acupuncture, especially EA, is generally considered a time-consuming treatment, a simpler acupuncture type, such as ear acupuncture, could be considered in large-scale PTSD events [14]. Our results show that this intervention has some preclinical evidence of the therapeutic mechanism, suggesting the potential to improve different clinical aspects of PTSD. Additionally, although not the outcome of interest in this review, other clinical effects of acupuncture, such as pain control, which may be important in PTSD patients, are well known. Therefore, the results of this study may help elucidate the possibility of using acupuncture for PTSD in terms of evidence-based medicine. However, the following limitations should be considered in this review. First, this review presents results based on animal studies, and it is not possible to directly correlate these results with therapeutic mechanisms of acupuncture in human PTSD patients. To our knowledge, few studies have investigated the therapeutic mechanisms of acupuncture in human PTSD subjects [13]. Therefore, the results of this review could be used as data for designing a future acupuncture mechanism study in PTSD patients. Second, although acupuncture was largely classified into EA and MA in this review, the difference in treatment sites, called acupoints, was not considered. Analysis of the frequency has provided some treatment site data, such as Baihui (GV 20), that may be important in future studies, so future studies will be able to investigate acupoint-specific effects on the main acupoints. Third, the methodological quality of the included animal studies was not high enough to produce robust evidence. The included studies were evaluated as unclear in most SYRCLE risk of bias items, and insufficient reporting was suggested as a major problem. Fourth, in the treatment of mental disorders, acupuncture is composed of a treatment procedure that includes a patient-practitioner interaction context, so some other effects, including expectancy effects, cannot be ignored [76,77]. Such effects of acupuncture are difficult to elucidate in animal studies; that is, it is suggested that there may be other therapeutic mechanisms that are not covered by the results of this review. Lastly, this systematic review does not have a preregistered protocol, reflecting a potential for reporting bias.

### 4.5. Future Direction

Considering the abovementioned limitations, future research in this field will be able to consider the following. First, research is needed to investigate the therapeutic mechanism of acupuncture on PTSD in humans. Ideally, tools, such as functional magnetic resonance imaging or functional near-infrared spectroscopy, can be used to investigate the therapeutic mechanism of acupuncture in terms of brain science. Moreover, in a few clinical studies, such an attempt has already been conducted [78]. The findings of this review may be used as a reference in the process. Second, by conducting clinical studies on some important acupoints proposed in the findings of this review, it may be possible to study acupoint-specific mechanisms or standardize the acupuncture method for treating PTSD. For example, scalp acupoints, such as Baihui (GV 20) and Shenting (GV 24), have traditionally been used to treat psychiatric disorders, and EA on Baihui (GV 20) and Shenting (GV 24) and other acupoints present in the head or neck, Shenting (GV 24), and Fengchi (GB 20), have been used in a clinical study to compare the effectiveness with that of paroxetine in the treatment of PTSD [79]. These acupoints may be promising in future research in this field. Third, it was found that most of the included studies were conducted in China. Although these studies were conducted on animal models, acupuncture methods, such as acupoint selection or stimulation method, in the study design stage may be influenced by the researchers. Thus, to reflect the acupuncture method uniquely used in countries other than China, it is necessary to conduct acupuncture studies on PTSD animal models in other countries as well. In addition, animal studies of acupuncture on PTSD need to be further improved, as current animal studies are insufficient to provide robust evidence on underlying mechanisms of acupuncture for PTSD.

## 5. Conclusions

This review summarizes the complex underlying therapeutic mechanisms of acupuncture, including MA and EA, in PTSD animal models. In behavioral evaluation, acupuncture, including MA and EA, reduced anxiety and fear responses and weakened fear conditioning, improved sleep architecture, reduced depressive symptoms, and alleviated the disturbance of spatial learning and memory of the PTSD animal models. The therapeutic mechanisms of acupuncture proposed in the included studies could be classified into two categories: (1) regulation of stress responses in the neuroendocrine system and (2) promotion of neuroprotection, neurogenesis, and synaptic plasticity in several brain areas. However, the methodological quality of the included animal studies was not high enough to produce robust evidence. In addition, mechanistic studies on specific aspects of acupuncture that may affect PTSD, including expectancy effects, in human PTSD subjects are also needed.

## Figures and Tables

**Table 1:** Characteristics of included studies.

Study	Species (Sex)	Age/Weight	PTSD Model Method	Main Outcome	Main Results Compared to MG (Acupuncture Group)
Fang 2012 [27]	SD rats (male)	NR/180–220 g	SPS	1. EPM (1) Time spent in open arms, (2) Entries in open arms 2. Serum corticosterone	1. (1) ↑ *, (2) ↑ ^†^ 2. ↓ *
Hou 2013a [28]	SD rats (male)	NR/220 ± 20 g	SPS	1. nNOS mRNA expression in hippocampus 2. nNOS protein expression in hippocampal (1) CA1 and (2) CA3 regions	1. ↓ * 2. (1) ↑ *, (2) ↑ *
Hou 2013b [29]	SD rats (male)	NR/220 ± 20 g	SPS	1. MWM (escape latency) 2. Protein expression in hippocampus (1) Glucocorticoid receptor, (2) Mineralocorticoid receptor 3. Ratio of mineralocorticoid receptor/glucocorticoid receptor expression	1. ↓ * 2. (1) ↓ *, (2) ↑ * 3. ↑ *
Li 2014 [30]	SD rats (male)	NR/180–220 g	Compound stress stimulation of restraint, electric shock, and exhaustive swimming	1. MWM (1) Escape latency, (2) Percent time in the III quadrant, (3) Crossing number in the III quadrant	1. (1) ↓ *, (2) ↑ *, (3) ↑ *
Xie 2015 [31]	SD rats (male)	NR/200–250 g	SPS	1. Behavioral observation (1) Number of grids, (2) Number of erection 2. Locus coeruleus nNOS positive cells 3. Mean gray value	1. (1) ↑ ^†^, (2) ↑ ^†^ 2. ↓ ^†^ 3. ↑ ^†^
Li 2016 [32]	SD rats (male)	NR/ 280–320 g	SPS	1. Freezing condition (freezing time) 2. EPM (1) Time spent in open arms, (2) Entries in open arms 3. Protein expression in hippocampus (1) Ask-1, (2) Bax, (3) Trx	1. ↓ * 2. (1) ↑ ^†^, (2) ↑ ^†^ 3. (1) ↓ *, (2) ↓ *, (3) ↑ *
Li 2017 [33]	SD rats (male)	8 weeks/250 ± 20 g	SPS&S	1. OFT (1) Entries in center, (2) time spent in center 2. EPM (Time spent in open arms) 3. mRNA expression in hippocampus (1) Sirt1, (2) MAO-A	1. (1) ↑ ^†^, (2) ↑ * 2. ↑ * 3. (1) ↓ ^†^, (2) ↓ *
Ding 2018 [34]	SD rats (male)	NR/NR	SPS&S	1. Scene conditioned fear response detection (1) Memory acquisition, (2) Memory extinction, (3) Memory reconstruction 2. Cue conditioned fear response detection (1) Memory acquisition, (2) Memory extinction, (3) Memory reconstruction 3. Protein expression in amygdala (1) BDNF, (2) TrkB, (3) p-ERK, (4) p-MEK, (5) p-ERK1/2	1. (1) N.S., (2) ↓ ^†^, 3) ↓ ^†^ 2. (1) N.S. or ↓ * or ↓ ^†^, (2) ↓ ^†^, (3) ↓ ^†^ 3. (1) ↑ ^†^, (2) ↑ ^†^, (3) ↑ ^†^, (4) ↑ ^†^, (5) ↑ ^†^
Chen 2019 [35]	SD rats (male)	8 weeks/220 ± 20 g	Enhanced SPS	1. OFT (1) Time spent in center, (2) Entries in center 2. EPM (1) Time spent in open arms, (2) Entries in open arms 3. Expression in prefrontal lobe (1) Endocannabinoid receptor type 1, (2) Monoacylglycerol lipase, (3) Diacylglycerol lipase	1. (1) N.S., (2) ↑ * 2. (1) ↑ *, (2) ↑ * 3. (1) ↑ *, (2) ↑ *, (3) N.S.
Li 2019 [36]	SD rats (male)	2 months/180–220 g	MG1: SPS MG2: SPS&S	1. Radial six-arm water maze test (1) Escape latency, (2) Distance travelled 2. Locomotor activity (1) Distance, (2) Shock intensity 3. EPM (1) Time spent in open arms, (2) Entries in open arms 4. Assessment of conditional fear response (1) Memory acquisition, (2) Memory extinction 5. PSD thickness (1) Amygdala, (2) Hippocampus 6. Synaptic gap width (1) Amygdala, (2) Hippocampus 7. Curvature of synaptic interfact (1) Amygdala, (2) Hippocampus 8. BDNF levels (1) Amygdala, (2) Hippocampus 9. fEPSP amplitude of hippocampus 10. Protein expression in amygdala (1) SYN, (2) GAP43, (3) PSD95 11. mRNA expression in amygdala (1) SYN, (2) GAP43, (3) PSD95 12. Protein expression in hippocampus (1) SYN, (2) GAP43, (3) PSD95 13. mRNA expression in hippocampus (1) SYN, (2) GAP43, (3) PSD95	MG1 + acupuncture, MG2 + acupuncture 1. (1) ↑ ^†^, ↑ ^†^, (2) ↑ ^†^, ↑ ^†^ 2. (1) ↑ * or ↑ ^†^, ↑ * or ↑ ^†^, (2) N.S., N.S. 3. (1) ↑ ^†^, ↑ ^†^, (2) ↑ ^†^, ↑ ^†^ 4. (1) N.S. or ↓ * or ↓ ^†^, N.S. or ↓ * or ↓ ^†^, (2) ↓ ^†^, ↓ ^†^ 5. (1) ↑ *, ↑ *, (2) ↑ *, ↑ ^†^ 6. (1) N.S., N.S., (2) N.S., ↓ * 7. (1) N.S., ↑ *, (2) ↑ ^†^, ↑ ^†^ 8. (1) ↑ *, ↑ ^†^, (2) ↑ ^†^, ↑ ^†^ 9. *p*-value was not presented 10. (1) ↑ ^†^, ↑ ^†^, (2) ↑ ^†^, ↑ ^†^, (3) ↑ ^†^, ↑ ^†^ 11. (1) ↑ ^†^, ↑ ^†^, (2) ↑ ^†^, ↑ ^†^, (3) ↑ ^†^, ↑ ^†^ 12. (1) ↑ ^†^, ↑ ^†^, (2) ↑ ^†^, ↑ ^†^, (3) ↑ ^†^, ↑ ^†^ 13. (1) ↑ ^†^, ↑ ^†^, (2) ↑ ^†^, ↑ ^†^, (3) ↑ ^†^, ↑ ^†^
Liu 2019 [37]	SD rats (male)	NR/180–220 g	SPS	1. OFT (1) Time spent in center, (2) Total distance 2. EPM (time spent in open arms) 3. Serum corticosterone 4. Fos-positive nuclei in the anterior cingulate cortex	1. (1) ↑ ^†^, (2) N.S. 2. ↑ * 3. ↓ * 4. ↑ *
Wei 2019 [38]	SD rats (male)	NR/250 ± 10 g	SPS	1. REMS 2. Slow wave sleep stage 1 3. Slow wave sleep stage 2 4. Levels in hypothalamus (1) IL-1β, (2) TNF-α, (3) IL-4, (4) IL-10	1. ↑ * 2. N.S. 3. ↑ * 4. (1) N.S., (2) ↓ *, (3) ↓ *, (4) N.S.
Xue 2019 [39]	SD rats (male)	NR/280–320 g	Enhanced SPS	1. OFT (1) Total distance, (2) Time spent in center, (3) Distance traveled in center 2. EPM (1) Time spent in open arms, (2) Entries in open arms, (3) distance traveled in open arms 3. Fear conditioning test (1) contextual freezing time, (2) cued freezing time 4. Protein expression in hippocampus (1) BDNF, (2) PSD95, (3) Syn, (4) CB1R, (5) DAGLα	1. (1) N.S., (2) ↑ *, (3) ↑ * 2. (1) ↑ *, (2) ↑ *, (3) ↑ * 3. (1) ↓ *, (2) ↓ * 4. (1) ↑ *, (2) ↑ *, (3) ↑ *, (4) ↑ *, (5) ↑ ^†^
Zhou 2019 [40]	SD rats (male)	NR/280–320 g	Enhanced SPS	1. OFT (1) Total distance, (2) Time spent in center 2. EPM (1) Time spent in open arms, (2) Entries in open arms 3. Fear conditioning test (1) contextual freezing time, (2) cued freezing time 4. Number of cells in dentate gyrus (1) BrdU ^†^ cells, (2) DCX ^†^ cells, (3) NeuN ^†^/Nrf2 ^†^ cells, (4) GFAP ^†^/Nrf2 ^†^ cells 5. Ratio of DCX/β-actin 6. Ratio of BDNF/β-actin 7. Ratio of pAMPKα/AMPKα 8. Ratio of Nrf2/β-actin 9. Ratio of HO-1/β-actin	1. (1) N.S., (2) ↑ * 2. (1) ↑ *, (2) ↑ * 3. (1) ↓ *, (2) ↓ * 4. (1) ↑ *, (2) ↑ *, (3) ↑ *, (4) ↑ * 5. ↑ * 6. ↑ * 7. ↑ * 8. ↑ ^†^ 9. ↑ ^†^
Zhu 2019 [41]	SD rats	NR/NR	SPS	1. Number in amygdala (1) BDNF positive neuron, (2) Tyrosine hydroxylase positive fiber	1. (1) ↓ ^†^, (2) ↓ ^†^
Zhao 2016 [42]	SD rats (male)	2 month old/180–220 g	SPS & S	Experiment (A) 1. Non-REMS latency (min) for 12 h in daytime 2. REMS latency (min) for 12 h in daytime 3. Awakening period (min) for 12 h in daytime 4. Total sleep time (min) for 12 h in daytime Experiment (B) 1. Action potential release of hippocampal 1) CA1 and 2) CA3 regions 2. Discharge frequency of hippocampal 1) CA1 and 2) CA3 regions (Hz) 3. Wave amplitude of hippocampal 1) CA1 and 2) CA3 regions (μV) 4. Interspike interval of hippocampal 1) CA1 and 2) CA3 regions 5. Power spectral densities of hippocampal 1) CA1 and 2) CA3 regions	Experiment (A) 1. ↓ ^†^ 2. ↓ * 3. ↓ ^†^ 4. ↑ ^†^ Experiment (B) 1. (1) ↑ ^†^, (2) ↑ ^†^ 2. (1) ↑ ^†^, (2) ↑ ^†^ 3. (1) ↑ ^†^, (2) ↑ ^†^ 4. (1) ↓ *, (2) ↓ ^†^ 5. (1) ↑ *, (2) ↑ ^†^
Han 2017 [43]	SD rats (male)	6 weeks old/180 ± 20 g	SPS&S	1. OFT (1) Horizontal crossing grid number, (2) Vertical frequency, (3) Number of fecal particle 2. MWM (1) Daily latency during positioning navigation experiment (sec), (2) Space exploration experiment-times crossing platform, (3) Space exploration experiment-times crossing the effective areas 3. New object recognition test (discrimination index) 4. EEG power spectrum values of bilateral cerebral hemispheres (left/right) (1) α-wave, (2) β-wave, (3) δ’-wave, (4) θ-wave	1. (1) ↑ ^†^, (2) ↑ ^†^, (3) ↓ ^†^ 2. (1) ↓ ^†^, (2) ↑ ^†^, (3) ↑ ^†^ 3. ↑ ^†^ 4. (1) ↑ ^†^, (2) ↓ ^†^, (3) ↓ ^†^, (4) ↓ ^†^
Oh 2018 [44]	SD rats (male)	8 weeks old/200–220 g	SPS	Experiment (A) 1. FST (1) Immobility time (s), (2) Climbing time (s), (3) Swimming time (s) 2. OFT (1) Entries in center, (2) Travel distance (cm) 3. EPM (1) Entries in open arms, (2) Entries in closed arms, (3) Time spent in open arms (open/total), (4) Anxiety index 4. Corticotrophin-releasing factor expression levels in the paraventricular nucleus of hypothalamus by immunofluorescence 5. Serum corticosterone (pg/mL) 6. Protein expression levels in the hippocampus (1) Extracellular signal-regulated kinase, (2) Akt, (3) mTOR, (4) p70S6K, (5) 4E-BP-1, (6) CREB 7. Synaptic protein expression levels in the hippocampus (1) PSD95, (2) Syn1, (3) GluR1, (4) BDNF Experiment (B) 1. FST (1) Immobility time (s), (2) Climbing time (s), (3) Swimming time (s) 2. OFT (1) Entries in center, (2) Travel distance (cm) 3. EPM (1) Entries in open arms, (2) Entries in closed arms, (3) Time spent in open arms (open/total), (4) Anxiety index	Experiment (A) 1. (1) ↓ ^†^, (2) ↑ ^†^, (3) N.S. 2. (1) ↑ ^†^, (2) N.S. 3. (1) ↑ ^†^, (2) N.S., (3) ↑ ^†^, (4) ↑ ^†^ 4. ↓ ^†^ 5. ↓ ^†^ 6. (1) N.S., (2) ↑ ^†^, (3) ↑ *, (4) ↑ ^†^, (5) ↑ ^†^, (6) ↑ ^†^ 7. (1) ↑ ^†^, (2) ↑ ^†^, (3) ↑ *, (4) ↑ ^†^ Experiment (B) 1. (1) ↓ ^†^, (2) ↑ ^†^, (3) N.S. 2. (1) ↑ ^†^, (2) ↑ * 3. (1) ↑ ^†^, (2) ↑ *, (3) ↑ ^†^, (4) ↑ ^†^
Wei 2018 [45]	SD rats (male)	6 weeks old/180 ± 20 g	SPS & S	1. MWM (1) Daily latency during positioning navigation experiment (sec), (2) Space exploration experiment-times crossing platform, 3) Space exploration experiment-times crossing the effective areas 2. EEG power spectrum values of bilateral cerebral hemispheres (left/right) (1) α-wave, (2) β-wave, (3) δ’-wave, (4) θ-wave	1. (1) ↓ *, (2) ↑ *, (3) ↑ * 2. (1) ↑ ^†^, (2) ↓ ^†^, (3) ↓ ^†^, (4) ↓ ^†^
Zhang 2018 [46]	SD rats (male)	6 weeks old/180 ± 20 g	SPS & S	1. fNIRs (1) Oxy-Hb, (2) Deoxy-Hb, (3) Total-Hb	1. (1) ↓ *, (2) ↑ *, (3) ↓ *
Zhao 2018 [47]	SD rats (male)	6 weeks old/180 ± 20 g	SPS & S	1. OFT (1) Horizontal crossing grid number, (2) Vertical frequency, (3) Number of fecal particle 2. MWM (1) Location navigation (escape latency), (2) Special probe tests (platform quadrant crossing times) 3. Discrimination index	1. (1) ↑ *, (2) ↑ *, (3) ↓ * 2. (1) ↓ *, (2) ↑ * 3. ↑ *
Zhao 2018 [48]	SD rats (male)	2 month old/200 ± 20 g	SPS & S	1. Discharge frequency of hippocampal (1) CA1 and (2) CA3 regions (Hz) 2. Wave amplitude of hippocampal (1) CA1 and (2) CA3 regions (μV)	1. (1) ↑ ^†^, (2) ↑ ^†^ 2. (1) ↑ ^†^, (2) ↑ ^†^
Yu 2019 [49]	Wistar rats (male)	8 weeks old/203.47 ± 6.29 g	SPS & S	1. Non-REMS latency (min) 2. REMS latency (min) 3. Amplitude and ultrastructure of the neurons in the hippocampal (1) CA1 and (2) CA3 regions	1. ↓ * 2. ↓ * 3. (1) ↑ *, (2) ↑ *
Zhao 2019 [50]	SD rats (male)	2 month old/200 ± 20 g	SPS & S	1. Interspike interval of hippocampal (1) CA1 and (2) CA3 regions 2. Power spectral densities of hippocampal (1) CA1 and (2) CA3 regions	1. (1) ↓ *, (2) ↓ ^†^ 2. (1) ↓ *, (2) ↓ ^†^

‘*’ and ‘^†^’ mean significant differences between two groups, *p* < 0.05 and *p* < 0.01. ‘N.S’. means no significant difference between two groups, *p* > 0.05. Abbreviations. ACC, anterior cingulate cortex; AMPK, adenosine monophosphate activated protein kinase; Ask-1, apoptosis signal-regulating kinase 1; Akt, protein kinase B; BDNF, brain-derived neurotrophic factor; BG, blank group; BrdU, bromodeoxyuridine; CB1R, cannabinoid type 1 receptor; CREB, cAMP response element-binding protein; DAGLα, diacylglycerol lipase α; DCX, doublecortin; EEG, electroencephalogram; EPM, elevated plus-maze; fEPSP, field excitatory postsynaptic potential; fNIRS, functional near-infrared spectroscopy; FST, forced swim test; GAP, growth-associated protein; GFAP, glial fibrillary acidic protein; HO-1, heme oxygenase-1; IL, interleukin; MAO-A, monoamine oxidase A; MG, model group; mRNA, messenger ribonucleic acid; mTOR, mammalian target of rapamycin; MWM, Morris water maze; NeuN, neuronal nuclei; nNOS, neuronal nitric oxide synthase; NR, not recorded; Nrf2, nuclear factor erythroid 2-related factor 2; OFT, open field test; SD, Sprague Dawley; SPS, single-prolonged stress; SPS & S, single-prolonged stress accompanied shock; SYN, synaptophysin; p-ERK, phosphorylated extracellular signal-regulated kinase; p-MEK, phosphorylated mitogen-activated protein/extracellular signal-regulated kinase kinase; PSD, postsynaptic density; PTSD, post-traumatic stress disorder; REMS, rapid eye movement sleep; Sirt1, Sirtuin 1; TNF, tumor necrosis factor; Trx, thioredoxin; TrkB, tropomyosin-related kinase B.

**Table 2:** Target symptoms and proposed mechanisms.

Study	Target Symptom	Proposed Mechanism
**Electro-Acupuncture**		
Fang 2012 [27]	Anxiety symptom	Downregulation of HPA axis activity
Hou 2013a [28]	Spatial learning and memory *	Downregulation of hippocampal nNOS expression
Hou 2013b [29]	Spatial learning and memory	Upregulation of hippocampal mineralocorticoid receptor expression Downregulation of hippocampal glucocorticoid receptor expression
Li 2014 [30]	Spatial learning and memory	NR
Xie 2015 [31]	Excessive stress response *	Downregulation of nNOS expression in locus coeruleus
Li 2016 [32]	Anxiety symptom	Upregulation of hippocampal Trx expression (relieve the nerve injury)
Li 2017 [33]	Conditioned fear	Restoration of the mRNA level of Sirt1 and MAO-A in the hippocampus
Ding 2018 [34]	Anxiety symptom	Upregulation of BDNF, TrkB, p-MEK, and p-ERK expression in the amygdala Activating BDNF-TrkB-ERK signaling pathway
Chen 2019 [35]	Anxiety symptom	Increasing CB1R and DAGLα expression in the PFC
Li 2019 [36]	Fear memory	Repair of synaptic plasticity in amygdala and hippocampus
Liu 2019 [37]	Fear memory	Increasing Fos expression in the ACC
Wei 2019 [38]	Sleep disturbance	Regulation TNF-α and IL-4 levels in the hypothalamus
Xue 2019 [39]	Anxiety symptom and fear learning	Improvement of hippocampal synaptic plasticity Increasing BDNF, DAGLα, and CB1R expression
Zhou 2019 [40]	Anxiety symptom	Improvement of hippocampal neurogenesis Upregulation of the molecular mechanism associated with protection against oxidative damage and of BDNF expression
Zhu 2019 [41]	Fear learning *	Prevention TH from increasing and BDNF from decreasing in amygdala
**Manual Acupuncture**		
Zhao 2016 [42]	Sleep disturbance	Restoring the hippocampal neural structure
Han 2017 [43]	Spatial learning and memory	Improvement of the abnormal EEG power spectrum value
Oh 2018 [44]	Depression and anxiety symptom	Increasing protein synthesis required for synaptic plasticity via the mTOR pathway in the hippocampus
Wei 2018 [45]	Spatial learning and memory	Improvement of the abnormal EEG activity
Zhang 2018 [46]	Not specific (abnormal neuron activity) *	Regulatory effect on the blood-oxygen concentration in cerebral cortex
Zhao 2018 [47]	Anxiety and learning-memory ability	NR
Zhao 2018 [48]	Fear memory *	Regulation of abnormal neuron cluster electrical activity of hippocampal CA1 and CA3 regions.
Yu 2019 [49]	Sleep disturbance	Reversing the discharge activity of neurons in hippocampus and restoring the structure of neuron cells
Zhao 2019 [50]	Excessive stress response *	Regulation of the neuro-electrophysiology activity of hippocampal CA1 and CA3 regions

**Abbreviations**. ACC, anterior cingulate cortex; BDNF, brain-derived neurotrophic factor; CB1R, cannabinoid type 1 receptor; DAGLα, diacylglycerol lipase α; DCX, doublecortin; EEG, electroencephalogram; HPA, hypothalamic-pituitary-adrenal; IL, interleukin; MAO-A, monoamine oxidase A; mRNA, messenger ribonucleic acid; mTOR, mammalian target of rapamycin; nNOS, neuronal nitric oxide synthase; NR, not recorded; p-ERK, phosphorylated extracellular signal-regulated kinase; PFC, prefrontal cortex; p-MEK, phosphorylated mitogen-activated protein/extracellular signal-regulated kinase kinase; Sirt1, Sirtuin 1; TH, tyrosine hydroxylase; TNF, tumor necrosis factor; Trx, thioredoxin; TrkB, tropomyosin-related kinase B. *, The studies did not perform behavioral test.

## Data Availability

The data used to support the findings of this study are included in the article.

## Appendix A

**Table A1:** PRISMA Checklist.

Section/Topic	#	Checklist Item	Reported on Page #
**TITLE**			
Title	1	Identify the report as a systematic review, meta-analysis, or both.	1
**ABSTRACT**			
Structured summary	2	Provide a structured summary including, as applicable: background; objectives; data sources; study eligibility criteria, participants, and interventions; study appraisal and synthesis methods; results; limitations; conclusions and implications of key findings; systematic review registration number.	1
**INTRODUCTION**			
Rationale	3	Describe the rationale for the review in the context of what is already known.	2
Objectives	4	Provide an explicit statement of questions being addressed with reference to participants, interventions, comparisons, outcomes, and study design (PICOS).	2
**METHODS**			
Protocol and registration	5	Indicate if a review protocol exists, if and where it can be accessed (e.g., Web address), and, if available, provide registration information including registration number.	NA
Eligibility criteria	6	Specify study characteristics (e.g., PICOS, length of follow-up) and report characteristics (e.g., years considered, language, publication status) used as criteria for eligibility, giving rationale.	3
Information sources	7	Describe all information sources (e.g., databases with dates of coverage, contact with study authors to identify additional studies) in the search and date last searched.	3
Search	8	Present full electronic search strategy for at least one database, including any limits used, such that it could be repeated.	Appendix A
Study selection	9	State the process for selecting studies (i.e., screening, eligibility, included in systematic review, and, if applicable, included in the meta-analysis).	3
Data collection process	10	Describe method of data extraction from reports (e.g., piloted forms, independently, in duplicate) and any processes for obtaining and confirming data from investigators.	3
Data items	11	List and define all variables for which data were sought (e.g., PICOS, funding sources) and any assumptions and simplifications made.	3
Risk of bias in individual studies	12	Describe methods used for assessing risk of bias of individual studies (including specification of whether this was done at the study or outcome level), and how this information is to be used in any data synthesis.	3
Summary measures	13	State the principal summary measures (e.g., risk ratio, difference in means).	3–4
Synthesis of results	14	Describe the methods of handling data and combining results of studies, if done, including measures of consistency (e.g., I^2^) for each meta-analysis.	NA
Risk of bias across studies	15	Specify any assessment of risk of bias that may affect the cumulative evidence (e.g., publication bias, selective reporting within studies).	NA
Additional analyses	16	Describe methods of additional analyses (e.g., sensitivity or subgroup analyses, meta-regression), if done, indicating which were pre-specified.	NA
**RESULTS**			
Study selection	17	Give numbers of studies screened, assessed for eligibility, and included in the review, with reasons for exclusions at each stage, ideally with a flow diagram.	4, Figure 1
Study characteristics	18	For each study, present characteristics for which data were extracted (e.g., study size, PICOS, follow-up period) and provide the citations.	4–5, Table 1, Table 2
Risk of bias within studies	19	Present data on risk of bias of each study and, if available, any outcome level assessment (see item 12).	13, Figure 2
Results of individual studies	20	For all outcomes considered (benefits or harms), present, for each study: (a) simple summary data for each intervention group (b) effect estimates and confidence intervals, ideally with a forest plot.	14–16
Synthesis of results	21	Present results of each meta-analysis done, including confidence intervals and measures of consistency.	NA
Risk of bias across studies	22	Present results of any assessment of risk of bias across studies (see Item 15).	NA
Additional analysis	23	Give results of additional analyses, if done (e.g., sensitivity or subgroup analyses, meta-regression [see Item 16]).	NA
**DISCUSSION**			
Summary of evidence	24	Summarize the main findings including the strength of evidence for each main outcome; consider their relevance to key groups (e.g., healthcare providers, users, and policy makers).	16–19
Limitations	25	Discuss limitations at study and outcome level (e.g., risk of bias), and at review-level (e.g., incomplete retrieval of identified research, reporting bias).	20
Conclusions	26	Provide a general interpretation of the results in the context of other evidence, and implications for future research.	21
**FUNDING**			
Funding	27	Describe sources of funding for the systematic review and other support (e.g., supply of data); role of funders for the systematic review.	21

*From:* Moher, D., Liberati, A., Tetzlaff, J., Altman, D.G., The PRISMA Group (2009). Preferred Reporting Items for Systematic Reviews and Meta-Analyses: The PRISMA Statement. PLoS Med 6(7): e1000097. doi:10.1371/journal.pmed1000097. For more information, visit: www.prisma-statement.org.

## Appendix B. Search Terms Used in Each Database

**Table A2:** Medline via PubMed.

	Searches	Results
#1	(“Trauma and Stressor Related Disorders” [MeSH] OR “posttraumatic stress disorder” [Title/abstract] OR PTSD [Title/abstract] OR trauma [Title/abstract] OR posttrauma [Title/abstract] OR posttraumatic [Title/abstract])	271346
#2	(“Acupuncture Therapy” [MeSH] OR “Acupuncture, Ear” [MeSH] OR “Acupuncture Points” [MeSH] OR “Acupuncture” [MeSH] OR “Electroacupuncture” [MeSH] OR “Meridians” [MeSH] OR acupuncture [Title/abstract] OR electroacupuncture [Title/abstract] OR electro-acupuncture [Title/abstract] OR acupoint * [Title/abstract])	30617
#3	(“Models, Animal” [MeSH] OR “animal” [ALL] OR “model” [ALL])	3183477
#4	#1 AND #2 AND #3	**39**

**Table A3:** EMBASE via Elsevier.

	Searches	Results
#1	(‘posttraumatic stress disorder’/exp OR ‘posttraumatic stress disorder’ OR ‘trauma’ OR ‘posttrauma’ OR ‘posttraumatic’ OR PTSD)	2435517
#2	(‘acupuncture’/exp OR ‘acupuncture’ OR ‘acupuncture therapy’ OR ‘auricular acupuncture’/exp OR ‘auricular acupuncture’ OR ‘ear acupuncture’ OR ‘acupuncture point’/exp OR ‘acupuncture point’ OR ‘electroacupuncture’/exp OR ‘electroacupuncture’ OR ‘electro-acupuncture’ OR ‘body meridian’/exp OR ‘body meridian’ OR ‘acupoint’)	52903
#3	(‘animal model’/exp OR ‘animal’ OR ‘model’)	8415222
#4	#1 AND #2 AND #3	**1206**

**Table A4:** AMED via EBSCO.

	Searches	Results
#1	(“Trauma and Stressor Related Disorders” [SU] OR “posttraumatic stress disorder” [TX] OR PTSD [TX] OR trauma [TX] OR posttrauma [TX] OR posttraumatic [TX])	4609
#2	(“Acupuncture Therapy” [SU] OR “Acupuncture, Ear” [SU] OR “Acupuncture Points” [SU] OR Acupuncture [SU] OR Electroacupuncture [SU] OR Meridians [SU] OR acupuncture [TX] OR electroacupuncture [TX] OR electro-acupuncture [TX] OR acupoint * [TX])	11403
#3	(“Models, Animal” [SU] OR animal [TX] OR model [TX])	31989
#4	#1 AND #2 AND #3	**4**

**Table A5:** CINAHL via EBSCO.

	Searches	Results
#1	(“Trauma and Stressor Related Disorders” [MH] OR “posttraumatic stress disorder” [TX] OR PTSD [TX] OR trauma [TX] OR posttrauma [TX] OR posttraumatic [TX])	227540
#2	(“Acupuncture Therapy” [MH] OR “Acupuncture, Ear” [MH] OR “Acupuncture Points” [MH] OR Acupuncture [MH] OR Electroacupuncture [MH] OR Meridians [MH] OR acupuncture [TX] OR electroacupuncture [TX] OR electro-acupuncture [TX] OR acupoint * [TX])	32495
#3	(“Models, Animal” [MH] OR animal [TX] OR model [TX])	1042824
#4	#1 AND #2 AND #3	**1068**

**Table A6:** PsycARTICLES via ProQuest.

	Searches	Results
#1	mesh (Trauma and Stressor Related Disorders) OR ‘posttraumatic stress disorder’ OR PTSD OR trauma OR posttrauma OR posttraumatic	20421
#2	mesh (Acupuncture Therapy) OR mesh (Acupuncture, Ear) OR mesh (Acupuncture Points) OR mesh (Acupuncture) OR mesh (Electroacupuncture) OR mesh (Meridians) OR ‘acupuncture’ OR ‘electroacupuncture’ OR ‘electro-acupuncture’ OR acupoint *	327
#3	mesh (Models, Animal) OR ‘animal’ OR ‘model’	121684
#4	#1 AND #2 AND #3	**118**

**Table A7:** OASIS.

	Searches	Results
#1	(외상 OR 트라우마 OR PTSD) AND 동물 AND 침	0
#2	(외상 OR 트라우마 OR PTSD) AND 모델 AND 침	0
#3	#1 OR #2	**0**

**Table A8:** KISS.

	Searches	Results
#1	(외상 OR 트라우마 OR PTSD) AND 동물 AND 침	7
#2	(외상 OR 트라우마 OR PTSD) AND 모델 AND 침	11
#3	#1 OR #2	**17**

**Table A9:** RISS.

	Searches	Results
#1	(외상 OR 트라우마 OR PTSD) AND 동물 AND 침	0
#2	(외상 OR 트라우마 OR PTSD) AND 모델 AND 침	1
#3	#1 OR #2	**1**

**Table A10:** KMbase.

	Searches	Results
#1	(트라우마 OR PTSD) AND (동물 OR 모델) AND 침	**8**

**Table A11:** KCI.

	Searches	Results
#1	(외상 OR 트라우마 OR PTSD) AND 동물 AND 침	1
#2	(외상 OR 트라우마 OR PTSD) AND 모델 AND 침	0
#3	#1 OR #2	**1**

**Table A12:** CNKI.

	Searches	Results
#1	(SU = ‘PTSD’+‘创伤后应激障碍’) AND (SU=‘acupuncture’+‘针’+‘鍼’) AND (SU=‘animal’+‘model’+‘动物’+‘模型’)	**11**

**Table A13:** Wanfang data.

	Searches	Results
#1	(主题: (“PTSD” + “创伤后应激障碍”) * 主题: (“acupuncture” + “针” + “鍼”) * 主题: (“animal” + “model” + “动物” + “模型”))	**126**

**Table A14:** VIP.

	Searches	Results
#1	(M = (PTSD OR 创伤后应激障碍)) AND (M = (acupuncture OR 针 OR 鍼)) AND (M = (animal OR model OR 动物 OR 模型))	**3**

**Table A15:** CiNii.

	Searches	Results
#1	(PTSD OR 创伤后应激障碍) AND (acupuncture OR 針 OR 鍼) AND (animal OR model OR 动物 OR 模型))	**1**

## Appendix C

**Table A16:** Funding Sources of Included Studies.

Studies	Funding Sources
Chen 2019	National Natural Science Foundation of China
Ding 2018	National Natural Science Foundation of China
Fang 2012	National Natural Science Foundation of China Sichuan Provincial Department of Education Natural Science Foundation Key Project
Hou 2013a	Anhui Natural Science Foundation Project Doctoral Research Fund of Wannan Medical College
Hou 2013b	Anhui Natural Science Foundation Project Doctoral Research Fund of Wannan Medical College
Li 2017	Shaanxi Province Science and Technology Rising Star Funded Project
Li 2019	National Natural Science Foundation of China
Li 2016	Not reported
Li 2014	Not reported
Liu 2019	Natural Science Foundation of China Science Research fund of Shanghai Municipal Commission of Health and Family The Three-Year development plan project for Traditional Chinese Medicine
Wei 2019	Hubei Province Traditional Chinese Medicine Integrated Traditional Chinese and Western Medicine Project High-level talent research projects in Xianning City, Hubei Province
Xie 2015	Anhui Province University Student Innovation Training Project
Xue 2019	National Natural Science Foundation of China National Science and technology support program of China the Fundamental Research Funds of Shaanxi Province
Zhou 2019	National Natural Science Foundation of China National Science and Technology Support Program of China the Fundamental Research Funds of Shaanxi Province
Zhu 2019	Provincial Excellent Young Talents Fund Project of Anhui Colleges and Universities Provincial Natural Science Foundation Project of Anhui Universities
Han 2017	2014 National Natural Science Foundation of China Regional Project and 2013 Gansu Provincial Natural Science Research Foundation Project
Oh 2018	the National Research Foundation of Korea funded by the Korean government (NRF-2015M3A9E3052338 and 2017R1A2B4009963) and the Korea Institute of Oriental Medicine
Wei 2018	Regional Science Fund of National Natural Science Foundation of China Natural Science Foundation of Gansu Province, China
Yu 2019	2015 Wuhan Clinical Medicine Research Project
Zhang 2018	Regional fund project of 2014 national natural science foundation of China 2013 Gansu provincial natural science foundation
Zhao 2018a	National Natural Science Foundation of China Gansu Province Natural Science Research Fund Project The 62nd batch of general projects of China Postdoctoral Science Foundation
Zhao 2016	National Natural Science Foundation of China Regional Projects and 2013 Gansu Provincial Natural Science Research Fund Projects
Zhao 2018b	National Natural Science Foundation of China Gansu Province Natural Science Research Fund Project General funded project of China Postdoctoral Science Foundation
Zhao 2019	General project of China postdoctoral science foundation Regional science fund of national natural science foundation of china 2013 natural science foundation of gansu province

## Appendix D

**Table A17:** Details of Acupuncture Treatment Methods.

Study	Acupuncture Methods	Acupoints	Course of Treatment	Treatment Duration
Fang 2012	EA, dilatational wave	Baihui (GV 20), Changqiang (GV 1)	15 min, once a day, total 3 sessions	3 days
Hou 2013a	EA, 2 Hz, 1 mA	Baihui (GV 20), Zusanli (ST 36)	30 min, once a day, total 7 sessions	1 week
Hou 2013b	EA, 2 Hz, 1 mA	Baihui (GV 20), Zusanli (ST 36)	30 min, once a day, total 7 sessions	1 week
Li 2014	EA, continuous wave, 2 Hz	Baihui (GV 20), Dazhui (GV 14)	20 min, once a day, total 10 sessions	10 days
Xie 2015	EA, 2 Hz, 3 V	Baihui (GV 20), Zusanli (ST 36)	20 min, once a day, total 7 sessions	1 week
Li 2016	EA, dilatational wave, 2/15 Hz, 1 mA	Baihui (GV 20)	30 min, once a day, total 5 sessions	1 week
Li 2017	EA, dilatational wave, 2/15 Hz, 1 mA	Baihui (GV 20)	30 min, once a day, total 7 sessions	1 week
Ding 2018	EA, dilatational wave, 2/100 Hz, 1 mA	Baihui (GV 20), Shenting (GV 24), Shenshu (BL 23)	20 min, once a day, total 21 sessions	3 weeks
Chen 2019	EA, dilatational wave, 2/15 Hz, 1 mA	Baihui (GV 20)	30 min, once a day, total 7 sessions	1 week
Li 2019	EA, dilatational wave, 2/100 Hz, 1 mA	Baihui (GV 20), Shenting (GV 24), Shenshu (BL 23)	20 min, once a day, total 21 sessions	3 weeks
Liu 2019	EA, constant current square wave, 2 Hz, 3 mA	Zusanli (ST 36)	20 min, once a day, total 7 sessions	1 week
Wei 2019	EA, 50/s	Baihui (GV 20)	20 min, once a day, total 7 sessions	1 week
Xue 2019	EA, dilatational wave, 2/15 Hz, 1 mA	Baihui (GV 20)	30 min, once a day, total 7 sessions	1 week
Zhou 2019	EA, dilatational wave, 2/15 Hz, 1 mA	Baihui (GV 20)	30 min, once a day, total 7 sessions	1 week
Zhu 2019	EA, continuous wave, 2 Hz	Baihui (GV 20), Zusanli (ST 36)	20 min, once a day, total 21 sessions	3 weeks
Zhao 2016 (same in experiment (A) and (B))	MA, basic manipulation for 1 min at each point	Baihui (GV 20), Neiguan (PC 6) *, Shenmen (HT 7) *, Taichong (LR 3) *	4 min, once a day, total 14 sessions	14 days
Han 2017	MA, small-amplitude twirling manipulation for 1 min at each point	Baihui (GV 20), Neiguan (PC 6) *, Shenmen (HT 7) *, Taichong (LR 3) *	4 min, once a day, total 12 sessions	12 days
Oh 2018 (same in experiment (A) and (B))	MA, turned at a rate of 2 spins/sec for 30 sec and removed immediately.	Shaofu (HT 8)	No retention, once a day, total 8 sessions	18 days
Wei 2018	MA, small-amplitude twirling manipulation for 1 min at each point	Baihui (GV 20), Neiguan (PC 6) *, Shenmen (HT 7) *, Taichong (LR 3) *	4 min, once a day, total 12 sessions	12 days
Zhang 2018	MA, minor lifting and thrusting and twirling for 1 min at each point	Baihui (GV 20), Neiguan (PC 6), Shenmen (HT 7), Taichong (LR 3)	4 min, once a day, total 12 sessions	12 days
Zhao 2018a	MA, minor lifting and thrusting and twirling for 1 min at each point	Baihui (GV 20), Neiguan (PC 6) *, Shenmen (HT 7) *, Taichong (LR 3) *	4 min, once a day, total 12 sessions	12 days
Zhao 2018b	MA, basic manipulation for 1 min at each point	Baihui (GV 20), Neiguan (PC 6) *, Shenmen (HT 7) *, Taichong (LR 3) *	4 min, once a day, total 14 sessions	2 weeks
Yu 2019	MA, manipulation for 1 min at each point	Baihui (GV 20), Neiguan (PC 6), Shenmen (HT 7), Taichong (LR 3)	4 min, once a day, total 7 sessions	1 week
Zhao 2019	MA, basic manipulation for 1 min at each point	Baihui (GV 20), Neiguan (PC 6) *, Shenmen (HT 7) *, Taichong (LR 3) *	4 min, once a day, total 14 sessions	2 weeks

* change left and right acupoint every day. Abbreviations. EA, electro-acupuncture; MA, manual acupuncture.

## Appendix E

**Table A18:** SYRCLE Results of Included Animal Studies.

Study	1	2	3	4	5	6	7	8	9	10
Fang 2012	U	U	U	U	U	N	U	U	Y	N
Hou 2013a	U	U	U	U	U	U	U	U	N	N
Hou 2013b	U	U	U	U	U	N	U	U	Y	Y
Li 2014	U	U	U	U	U	N	U	U	N	N
Xie 2015	U	U	U	U	U	U	U	U	Y	N
Li 2016	U	U	U	U	U	U	U	U	Y	N
Li 2017	U	U	U	U	U	N	U	U	Y	N
Ding 2018	U	U	U	U	U	U	Y	U	Y	Y
Chen 2019	U	U	U	U	U	U	U	U	Y	N
Li 2019	U	U	U	U	U	U	U	U	Y	Y
Liu 2019	U	U	U	Y	U	U	Y	U	Y	Y
Wei 2019	U	U	U	U	U	N	U	U	Y	Y
Xue 2019	U	U	U	U	U	U	U	U	Y	N
Zhou 2019	U	U	U	U	U	U	U	U	Y	N
Zhu 2019	U	U	U	U	U	U	U	U	N	Y
Zhao 2016 (experiment (A))	Y	U	U	U	U	U	U	N	Y	Y
Zhao 2016 (experiment (B))	Y	U	U	U	U	U	U	Y	Y	Y
Han 2017	Y	U	U	U	U	U	U	N	Y	Y
Oh 2018 (experiment (A))	U	U	U	Y	U	U	U	N	Y	Y
Oh 2018 (experiment (B))	U	U	U	Y	U	U	U	N	N	Y
Wei 2018	U	U	U	U	U	U	U	N	Y	Y
Zhang 2018	Y	U	U	N	U	Y	U	Y	N	Y
Zhao 2018a	U	U	U	U	U	U	U	Y	N	Y
Zhao 2018b	Y	U	U	Y	U	U	U	Y	N	Y
Yu 2019	Y	U	U	U	U	U	U	Y	Y	Y
Zhao 2019	Y	U	U	Y	U	U	U	Y	N	Y

1: selection bias (sequence generation), 2: selection bias (baseline characteristics), 3: selection bias (allocation concealment), 4: performance bias (random housing), 5: performance bias (blinding), 6: detection bias (random outcome assessment), 7: detection bias (blinding), 8: attrition bias (incomplete outcome data), 9: reporting bias (selective outcome reporting), 10: other sources of bias. “Y”, “N”, and “U” mean “Yes” (low risk of bias), “No” (high risk of bias), and “Unclear”, respectively.
